# Identification by TCGA database search of five genes that are aberrantly expressed and involved in hepatocellular carcinoma potentially via DNA methylation changes

**DOI:** 10.1186/s12199-020-00871-8

**Published:** 2020-07-23

**Authors:** Junya Matsushita, Takehiro Suzuki, Kazuyuki Okamura, Gaku Ichihara, Keiko Nohara

**Affiliations:** 1 Center for Health and Environmental Risk Research, National Institute for Environmental Studies, Tsukuba, Japan; 2 Graduate School of Pharmaceutical Sciences, Tokyo University of Science, Noda, Japan

**Keywords:** Hepatocellular carcinoma, DNA methylation, Gene expression, TCGA

## Abstract

**Background:**

Various treatments for hepatocellular carcinoma (HCC) are utilized in clinical practice; however, the prognosis is still poor on account of high recurrence rates. DNA methylation levels of CpG islands around promoters (promoter CpGis) inversely regulate gene expression and closely involved in carcinogenesis. As a new strategy, several chemicals globally inhibiting DNA methylation have been developed aiming at reducing DNA methylation levels and maintaining the expression of tumor suppressor genes. On the other hand, since these drugs nonspecifically modify DNA methylation, they can cause serious adverse effects. In order to ameliorate the methods by targeting specific CpGs, information of cancer-related genes that are regulated by DNA methylation is required.

**Methods:**

We searched candidate genes whose expressions were regulated by DNA methylation of promoter CpGi and which are involved in HCC cases. To do so, we first identified genes whose expression were changed in HCC by comparing gene expressions of 371 HCC tissues and 41 non-tumor tissues using the Cancer Genome Atlas (TCGA) database. The genes were further selected for poor prognosis by log-rank test of Kaplan-Meier plot and for cancer relevance by Pubmed search. Expression profiles of upregulated genes in HCC tissues were assessed by Gene Ontology (GO) analysis. Finally, using DNA methylation data of TCGA database, we selected genes whose promoter DNA methylation levels were inversely correlated with gene expression.

**Results:**

We found 115 genes which were significantly up- or downregulated in HCC tissues and were associated with poor prognosis and cancer relevance. The upregulated genes were significantly enriched in cell division, cell cycle, and cell proliferation. Among the upregulated genes in HCC, we identified hypomethylation of CpGis around promoters of *FANCB*, *KIF15*, *KIF4A*, *ERCC6L*, and *UBE2C*. In addition, TCGA data showed that the tumor suppressor gene *P16* is unexpectedly overexpressed in many types of cancers.

**Conclusions:**

We identified five candidate genes whose expressions were regulated by DNA methylation of promoter CpGi and associate with cancer cases and poor prognosis in HCC. Modification of site-specific DNA methylation of these genes enables a different approach for HCC treatment with higher selectivity and lower adverse effects.

## Background

Hepatocellular carcinoma (HCC) is the main type of liver cancer and has poor prognosis and low survival rates [1–3]. The GLOBOCAN database estimates that HCC is the sixth most commonly diagnosed cancer, and the fourth ranked contributor to the cause of cancer-related deaths [4]. Worldwide, approximately 841,000 new cancer cases and 782,000 deaths were reported to occur because of HCC in 2018 [4]. For inhibiting tumor-specific biological reactions, there are a variety of medical techniques, such as surgical resection, liver transplantation, and medication [2]. However, due to the high recurrence rates and metastasis, the prognosis of HCC patients is still poor, and the 5-year disease-free survival rate was 50.2% [4]. Hence, it is necessary to develop different approaches for the diagnosis and therapy of HCC.

Epigenetic modifications, such as DNA methylation, histone modifications, and non-coding RNAs (ncRNA) are pivotal factors of gene expression regulation without alteration of the DNA sequence [5]. Among them, DNA methylation is an extensively characterized epigenetic mechanism of gene regulation in mammals. DNA methylation levels of CpG islands (CpGis) around the transcription start sites (promoter CpGis) conversely regulate gene expression [6] and such alterations are closely involved in various cancers including HCC [7–9]. The tumor suppressor gene *P16* (cyclin-dependent kinase inhibitor 2A) is a well-known target that suffers DNA methylation silencing in human cancers [10, 11]. Suppression of DNA methylation changes at such specific targets are expected to provide new approaches to cancer treatment [8, 12].

Several drugs targeting aberrant DNA methylation, such as 5-aza-2′-deoxycytidine (5-aza-dC), have already been utilized for the therapy of refractory or relapsed cancer patients [12, 13]. 5-aza-dC exerts anticancer activity by effectively reducing DNA methylation levels and restoring the expression of *P16* [14, 15]. However, since this type of drug globally reduces DNA methylation, severe side effects were observed in clinical use [16]. To reduce this risk, it is desirable to restore the site-specific DNA methylation levels involved in cancer progression. For the purpose, recent techniques, such as the Crispr-Cas9 system, enable manipulation of DNA methylation/demethylation at specific sites [17–19]. On the other hand, fewer DNA methylation targets are identified in HCC compared to colon or gastric cancers [20].

The Cancer Genome Atlas (TCGA) database (https://tcga-data.nci.nih.gov/tcga/) provides valuable information about not only gene expression but also DNA methylation levels in various cancers from patients in multi-stages. Using the TCGA database, the present study aimed to identify DNA methylation changes which regulate the cancer-related gene expressions in HCC tissues to propose candidate targets for treatment.

For the purpose, we searched for cancer-related genes whose expression levels are significantly altered in HCC and which are associated with poor survival rates using data of 371 HCC tissues and 41 non-tumor tissues compiled in the TCGA database. They were further selected by the presence of promoter CpGis and significant changes of CpGi methylation levels in HCC tissues. Among them, we found 5 genes whose expressions are inversely correlated with DNA methylation and related to poor prognosis. Overall, we identified cancer-related genes whose expressions are associated with the DNA methylation of promoter CpGis in HCC tissues. The method described in this study is applicable to other types of cancers to identify candidates of genes that are regulated by DNA methylation.

## Materials and methods

### Gene expression and DNA methylation data for HCC and non-tumor tissues

Gene expression and DNA methylation data of HCC patients were downloaded from TCGA (https://cancergenome.nih.gov/) using UCSC Xena (http://xena.ucsc.edu/, version 10-04-2017). We selected total 371 HCC samples and 41 non-tumor samples which are opened for both DNA methylation and gene expression data. The clinical data of HCC patients are shown in Table 1 and sample ID lists are shown in Table S1. Gene Expression Profiling Interactive Analysis (GEPIA, http://gepia.cancer-pku.cn/) was utilized to obtain the expression data of *P16* in various cancers.

**Table 1:** Clinical features of patients

		Number of patients (rates)	
		Non-tumor tissue	HCC
Races	White	26 (63.4%)	184 (49.6%)
	American Indian or Alaska native	0 (0%)	2 (0.5%)
	Black or African American	7 (17.1%)	17 (4.6%)
	Asian	5 (12.2%)	158 (42.6%)
	Not reported	3 (7.3%)	10 (2.7%)
Age (year)		20–78	16–85
Gender	Male	18 (43.9%)	250 (67.4%)
	Female	23 (56.1%)	121 (32.6%)
History of HCC factor	None	14 (34.1%)	91 (24.5%)
	Alcohol consumption	6 (14.6%)	68 (18.3%)
	Hepatitis B	6 (14.6%)	75 (20.2%)
	Hepatitis C	5 (12.2%)	32 (8.6%)
	Non-alcoholic fatty liver disease	2 (4.9%)	11 (3.0%)
	Hemochromatosis	0 (0%)	5 (1.3%)
	Complex	1 (2.4%)	58 (15.6%)
	Other	4 (9.8%)	12 (3.2%)
	No data	3 (7.3%)	19 (5.1%)
Histologic grade	G1	5 (12.2%)	55 (14.8%)
	G2	20 (48.8%)	177 (47.7%)
	G3	14 (34.1%)	122 (32.9%)
	G4	2 (4.9%)	12 (3.2%)
	No data	0 (0%)	5 (1.3%)
AJCC TNM staging system^a^ (presence or absence of distant metastasis)	M0	27 (65.9%)	266 (71.7%)
	M1	1 (2.4%)	4 (1.1%)
	MX^b^	13 (31.7%)	101 (27.2%)
AJCC TNM staging system (lymph node involvement)	N0	25 (61.0%)	252 (67.9%)
	N1	1 (2.4%)	4 (1.1%)
	NX^c^	14 (34.1%)	114 (30.7%)
	No data	1 (2.4%)	1 (0.3%)
AJCC TNM staging system (tumor size and extent of tumors)	T1	19 (46.3%)	181 (48.8%)
	T2	10 (24.4%)	94 (25.3%)
	T3	9 (22.0%)	80 (21.6%)
	T4	3 (7.3%)	13 (3.5%)
	Other^d^	0 (0%)	3 (0.8%)
AJCC pathological stage	Stage I	17 (41.5%)	171 (46.1%)
	Stage II	7 (17.1%)	86 (23.2%)
	Stage III	8 (19.5%)	85 (22.9%)
	Stage IV	1 (2.4%)	5 (1.3%)
	No data^e^	8 (19.5%)	22 (5.9%)
	Other^e^	0 (0%)	2 (0.5%)

^a^AJCC TNM staging system: tumor-node-metastasis staging system which was creased and is updated by the American Joint Committee on Cancer (AJCC) (https://www.cancer.gov/about-cancer/diagnosis-staging/staging)

^b^MX: metastasis cannot be measured

^c^NX: cancer in nearby lymph nodes cannot be measured

^d^Other includes TX (main tumor cannot be measured), [discrepancy], and no data

^e^Other includes [discrepancy]

No data, data are not available

### Survival analysis

The relationships between the expression of 929 genes and overall survival time of HCC patients were found by a log-rank test of Kaplan-Meier analysis using OncoLnc (http://www.oncolnc.org/). In the analysis of OncoLnc, 360 HCC data were available. For the analysis of each gene, HCC patients (*n* = 360) were divided into the top 20% expression group (*n* = 72) and bottom 20% expression group (*n* = 72). Kaplan-Meier analysis for *P16* was also carried out using data of top and bottom 25% expression groups (*n* = 90). For the analysis of 5-year survival rate, survival rate data for the top and bottom 20% expression group were retrieved from TCGA using OncoLnc and analyzed using EZR (Saitama Medical Center, Jichi Medical University, Saitama, Japan, version 1.42) [21]. The hazard ratios (HRs) and 95% confidence intervals (CIs) of HCC were estimated with reference to the bottom 20% expression group using a Cox proportional hazard regression model that included several covariates (gender, age, history, histological grade, and AJCC pathological stage).

### Pubmed search

**Fig. 1:**
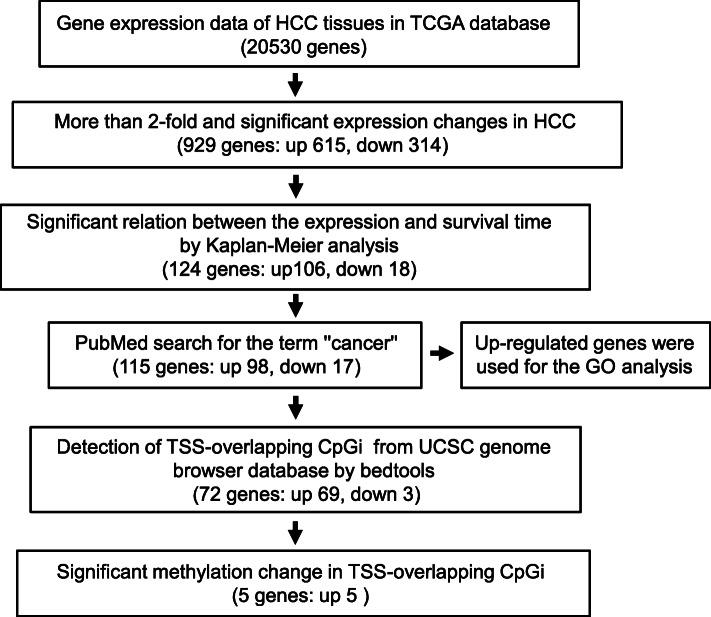
A flowchart of the gene selection process. This shows the steps which we chose the final 5 genes out of the TCGA database containing 20,530 genes

The cancer relevancy of the 124 genes shown in Fig. 1 was found by using the PubMed database (version 10-14-7) with the search term “cancer.” The search details were “gene” [All Fields] AND (“neoplasms”[MeSH Terms] OR “neoplasms”[All Fields] OR “cancer”[All Fields]).

### Identification of promoter CpGis and calculation of methylation value (*β* value)

The positions of CpGis and TSSs (transcription start sites) in the reference genome hg38 were obtained from the University of California, Santa Cruz (UCSC) genome browser website (https://genome.ucsc.edu/). The promoter CpGis that are overlapping TSSs were detected by bedtools 2.25.0 (bedtools.readthedocs.io) in the Cygwin64 software. The average CpGi methylation *β* value (methylated array intensity/(methylated + unmethylated array intensity)) of the top 20% expression-changed HCC (*n* = 74) and that of control livers (*n* = 41) were compared for each gene.

### Functional enrichment analysis using the Database for Annotation, Visualization and Integrated Discovery (DAVID)

Gene Ontology (GO) enrichment analysis including the biological process cellular component and molecular function was performed for upregulated genes shown in Table S2 by using DAVID v6.8 (https://david.ncifcrf.gov/) [22, 23].

### Statistical analysis

The differences in methylation *β* values of CpGs and gene expression levels between HCC tissues and non-tumor tissues were analyzed by a two-tailed paired Student’s *t* test. The methylation *β* values of CpGis were analyzed by a Wilcoxon rank sum test using an online program (http://www.gen-info.osaka-u.ac.jp/MEPHAS/wilc1.html). The *β* values of *P16* in HCC tissues of pathological stages I, II, III, and IV and non-tumor tissues were analyzed by one-way ANOVA followed by Turkey-Kramer test as a post hoc comparison. A *p* value < 0.05 was considered statistically significant. In the GO analysis, *p* values were controlled for false discovery rate (FDR) using the Benjamini-Hochberg test. *p* values of < 0.05 were considered statistically significant.

## Results

### Selection of cancer-related genes whose expressions were up- or downregulated and associated with poor prognosis in human hepatocellular carcinoma

To identify cancer-related genes whose expressions were inversely correlated with DNA methylation in HCC tissues, we analyzed data of 371 HCC tissues and 41 non-tumor tissues compiled in the TCGA database using the algorithm depicted in Fig. 1. The clinical features of 371 patients are summarized in Table 1. HCC is reported to be more common in males, and hepatitis B (HVB) infection is the most common etiological factor of HCC in the world. Hepatitis C virus (HVC) infection, alcohol abuse, and non-alcoholic fatty liver disease are other major risk factors [3, 24]. The clinical features shown in Table 1 were similar to the previous findings, although “none” was the most abundant feature in the history in the TCGA database.

After eliminating low expression genes by the criteria of expression values < 1.0, we selected 929 genes which are significantly up- or downregulated more than 2-fold in HCC tissues compared with non-tumor tissues.

For the 929 genes, we performed Kaplan-Meier survival analysis between the top 20% expression group and bottom 20% expression group, and found that 124 genes (106 and 18 genes up- or downregulated in HCC, respectively) have a significant relevance between overall survival time and the expression. From the 124 genes, we shortlisted 115 genes (98 upregulated and 17 downregulated in HCC) which appeared in a Pubmed search for the term “cancer” and “neoplasm” (Table S2).

### Upregulated genes in HCC were associated with cell cycle, cell division and cell proliferation

**Fig. 2:**
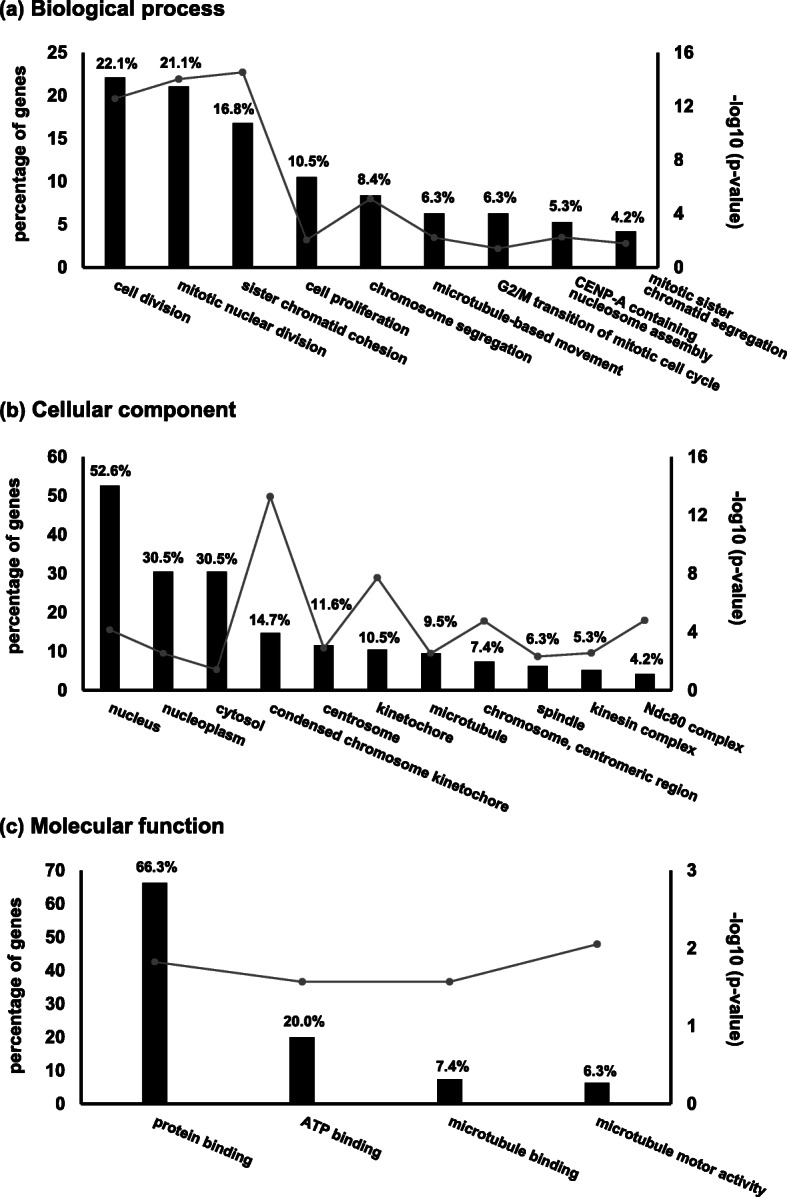
Gene Ontology enrichment analysis of the upregulate genes shown in Table S2. Bar graphs represent the percentages of genes involved in each term of biological process (BP) (**a**), cellular component (CC) (**b**), and molecular function (MF) (**c**). Line graphs represent the Benjamini corrected *p* values (significant difference, *p* < 0.05). Significantly enriched GO terms were shown in these graphs

To assess the roles of 98 genes that were upregulated more than 2-fold in HCC tissues (listed in Table S2), we performed GO analysis using the DAVID database. Nine out of 43 terms were significantly enriched in the field of biological processes as shown in Fig. 2a. The majority of the terms were related to cell division, cell cycle, and proliferation. In the field of cellular components (Fig. 2b), genes were particularly enriched in the terms related to the nucleus. In the molecular function field, the major parts of genes were categorized in the terms related to protein binding (Fig. 2c). We further found that 19 out of 98 genes were not only involved in cell division, cell cycle, or cell proliferation, but also located in the nucleus and had molecular functions of protein binding. We omitted GO analysis for downregulated genes due to the small number of them (17 genes listed in Table S2).

### Analysis of methylation status of promoter CpGis

Since DNA methylation levels in promoter CpGis are closely correlated with gene expression, we examined whether 115 genes shown in Table S2 have promoter CpGis and whether they are hypo- or hyper-methylated in HCC. We found that 72 out of 115 genes have CpGis overlapping TSSs (Fig. 1).

**Fig. 3:**
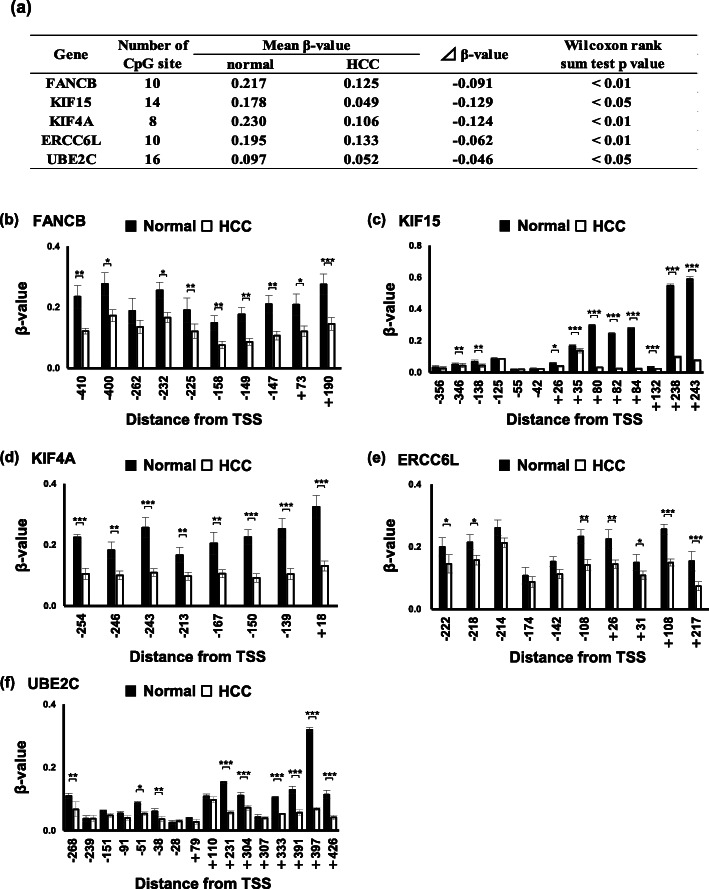
CpGis of *FANCB*, *KIF15*, *KIF4A*, *ERCC6L*, and *UBE2C* were hypomethylated in HCC tissues. **a** Mean methylation *β* values were calculated using data from 74 HCC tissues highly expressing each gene and data from 41 non-tumor tissues. Wilcoxon rank sum test was conducted using an online program and *p* value < 0.05 was considered statistically significant. The *β* values of CpG in CpGi of *FANCB* (**b**), *KIF15* (**c**), *KIF4A* (**d**), *ERCC6L* (**e**), and *UBE2C* (**f**) in 74 HCC tissues highly expressing each gene were compared with the *β* values of 41 non-tumor tissues. Statistical significance between the two groups was analyzed by Student’ s *t* test. *. **. *** Significantly different at *p* < 0.05, *p* < 0.01, and *p* < 0.001, respectively

We compared the top 20% mean *β* values of patients (*n* = 74) who showed higher or lower expression in HCC tissues and mean *β* values of non-tumor tissues (*n* = 41). Five genes, *FANCB* (FA complementation group B), *KIF15* (kinesin family member 15), *KIF4A* (kinesin family member 4A), *ERCC6L* (ERCC excision repair 6 like), and *UBE2C* (ubiquitin conjugating enzyme E2 C), were significantly hypomethylated in HCC tissues compared with non-tumor tissues (*p* value < 0.05 or 0.01, Fig. 3a). Furthermore, multiple CpG sites in the CpGis of these genes were significantly hypomethylated in HCC tissues (Fig. 3b–f).

### *FANCB*, *KIF15*, *KIF4A*, *ERCC6L*, and *UBE2C* were significantly upregulated and their expressions were associated with low survival rates in HCC

**Fig. 4:**
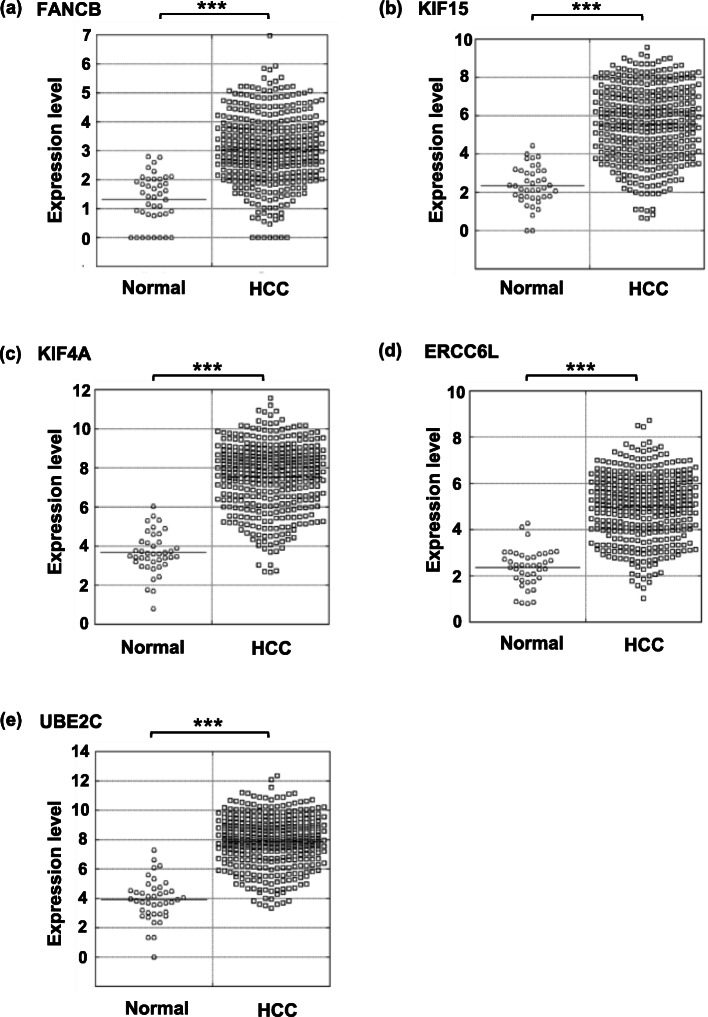
The expression levels of *FANCB* (**a**), *KIF15* (**b**), *KIF4A* (**c**), *ERCC6L* (**d**), and *UBE2C* (**f**). Statistical significance between the two groups was analyzed by Student’s *t* test. *** Significantly different at *p* < 0.001

The expression levels of *FANCB* (a), *KIF15* (b), *KIF4A* (c), *ERCC6L* (d), and *UBE2C* (e) in the individual 41 non-tumor tissues and 371 HCC tissues are plotted in Fig. 4. The expression of non-tumor tissues and HCC tissues were significantly different with *p* < 0.001 for all genes.

**Fig. 5:**
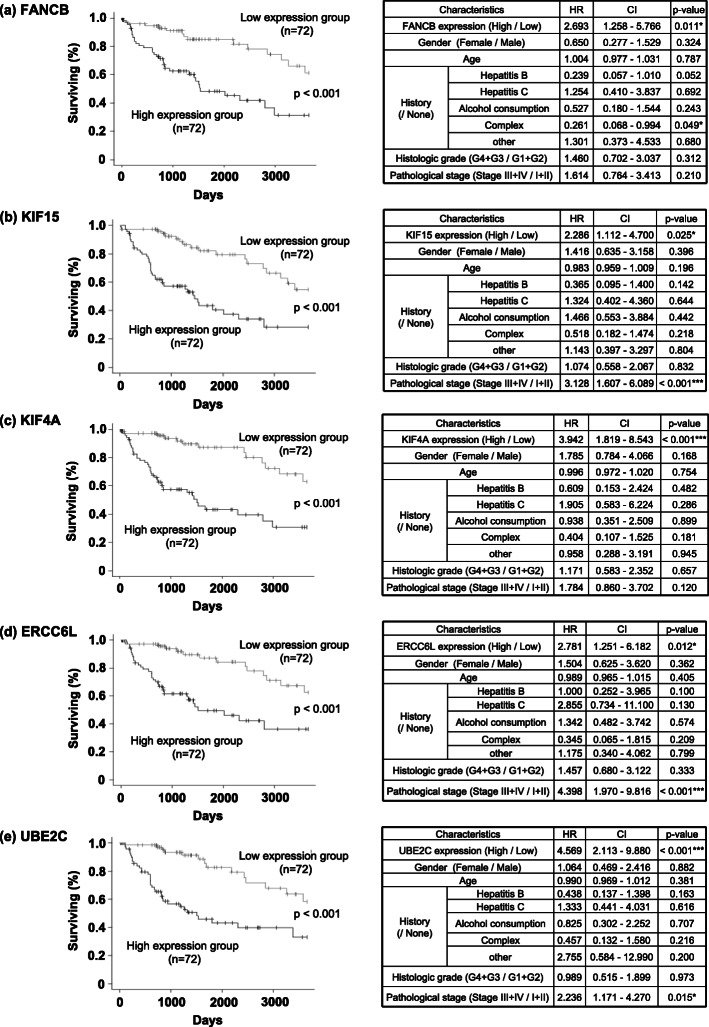
Kaplan-Meier analysis and Cox regression analysis of overall survival of *FANCB* (**a**), *KIF15* (**b**), *KIF4A* (**c**), *ERCC6L* (**d**), and *UBE2C* (**e**). Left figures and right tables were the results of Kaplan-Meier analysis and Cox regression analysis for 5-year survival data, respectively. Data from 72 HCC patients with higher or lower expression for each gene were selected in this study. A *p* value < 0.05 was considered statistically significant by log-rank test. In the Cox regression analysis, statistical significance is marked with the star symbol: *p < 0.05, **p < 0.01, ***p < 0.001. HR hazard ratio, CI confidence interval. Complex: including more than two histories. Other: including non-alcoholic fatty liver disease, hemochromatosis, and “other”

As the expression of these five genes were shown to be closely associated with poor prognosis in early time by Kaplan-Meier analysis as shown in the selection process of 124 genes, we focused on the 5-year survival rate of them. The results of Kaplan-Meier survival analysis for these five genes are shown in Fig. 5a–e (left panels). The prognosis of the higher expression groups of all the five genes were significantly poor compared with their low expression groups, and this poor prognosis persisted even after adjustment for several confounders (Fig. 5a–e, right panels). AJCC pathological stage was the significant covariate with the expression of *KIF15*, *ERCC6L*, and *UBE2C*. *FANCB* expression and gender were associated with poor prognosis.

### The CpGs of *P16* were hyper-methylated but upregulated in HCC tissues

**Fig. 6:**
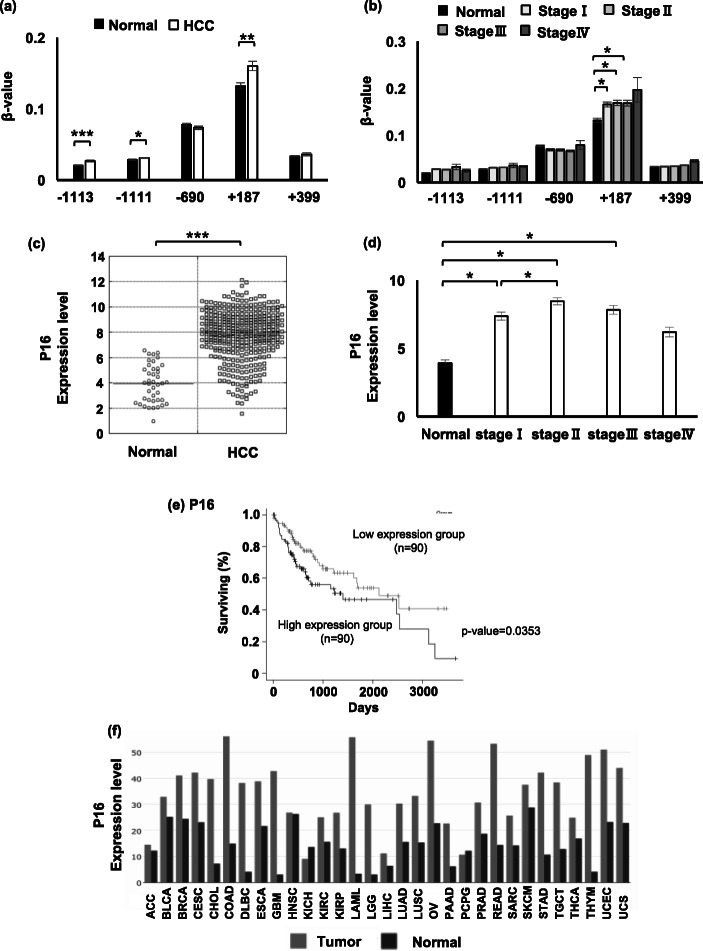
DNA methylation, gene expression, and survival analysis of *P16* gene. **a** The *β* values in the 74 HCC tissues highly expressing *P16* and in 41 non-tumor tissues. Statistical significance between the two groups was analyzed by Student’s *t* test. *. **. *** Significantly different at *p* < 0.05, *p* < 0.01, and *p* < 0.001 respectively. **b** The *β* values of *P16* in HCC tissues of pathological stages I, II, III, and IV and non-tumor tissues. Statistical significance was analyzed by one-way ANOVA followed by Turkey-Kramer test as a post hoc comparison. * Significantly different at *p* < 0.05. **c** The expression levels of *P16* in HCC tissues and non-tumor tissues. **d** The expression levels of *P16* in HCC tissues of pathological stages I, II, III, and IV and non-tumor tissues. **e** Kaplan-Meier analyzes for 90 HCC patients with higher and lower expression of *P16*. **f** The expression levels of *P16* in various types of cancers. Light black and dark black bars indicate expression in tumor tissues and non-tumor tissues, respectively. ACC adenoid cystic carcinoma, BLCA bladder carcinoma, BRCA breast invasive carcinoma, CESC cervical squamous cell carcinoma, CHOL cholangiocarcinoma, COAD colon adenocarcinoma, DLBC diffuse large B cell lymphoma, ESCA esophageal carcinoma, GBM glioblastoma, HNSC head-neck squamous cell carcinoma, KICH kidney chromophobe, KIRC kidney renal clear cell carcinoma, KIRP kidney renal papillary cell carcinoma, LAML acute myeloid leukemia, LGG low-grade glioma, LIHC liver hepatocellular carcinoma, LUAD lung adenocarcinoma, LUSC lung squamous cell carcinoma, OV ovarian cancer, PAAD pancreatic adenocarcinoma, PCPG pheochromocytoma and paraganglioma, PRAD prostate adenocarcinoma, READ rectum adenocarcinoma, SAC sarcoma, SKCM skin cutaneous melanoma, STAD stomach adenocarcinoma, TGCT testicular germ cell tumors, THCA thyroid cancer, THYM thymoma, UCEC uterine corpus endometrial carcinoma, UCS uterine carcinosarcoma

*P16* is well-recognized as a tumor suppressor gene which is silenced by hyper-methylation of CpGi in human cancers and restores expression by DNA methyltransferase inhibitors [10, 14, 25, 26]. We investigated the DNA methylation and gene expression of *P16* using the TCGA database. Consistent with previous studies, 3 CpG sites in CpGi were significantly hyper-methylated in HCC tissues (Fig. 6a). The methylation level of CpG site at + 187 bp from TSS significantly increased from the early stage of HCC (Fig. 6b). On the other hand, the expression of *P16* is upregulated in HCC tissues compared with non-tumor tissues (Fig. 6c) and the upregulation occurred from the early stage of HCC (Fig. 6d). Kaplan-Meier analysis revealed that the top 25% expression group of *P16* showed significant poor prognosis compared to the bottom 25% expression group in HCC (Fig. 6e). The prognosis of the higher and lower expression group was not significantly different by Cox regression analysis (data not shown). The results showed that *P16* is upregulated and is relevant to poor prognosis; however, its expression is not inversely correlated with DNA methylation in the HCC tissues. Further analysis showed that *P16* is upregulated in various cancer types including breast, colon, lung, and kidney (Fig. 6 f.).

## Discussion

In the present study, using the TCGA database, we searched for the candidates of cancer-related genes whose expressions are regulated by DNA methylation of CpGis and involved in poor prognosis in HCC tissues. We performed GO analysis for the 98 genes which are upregulated and associated with poor prognosis in HCC and are cancer relevant (Fig. 1). The result showed that they were significantly involved in the G2/M transition of the mitotic cell cycle, mitotic nuclear division, mitotic sister chromatid segregation, cell division, and cell proliferation (Fig. 2). These terms were related to cell malignancy, and their abnormalities or incompletions may lead to carcinogenesis [27–29]. In addition, more than half of the genes are located in the nuclei (Fig. 2b) where early events of cell division occur and mainly have a molecular function of protein binding, which suggest that they belong to transcription regulators controlling cell division and proliferation.

Among the 98 genes, we identified five genes (*FANCB*, *KIF15*, *KIF4A*, *ERCC6L*, and *UBE2C*) whose expressions were inversely correlated with DNA methylation (Figs. 3, 4, and 5). Recently, Sun et al. reported on the correlation analysis between DNA methylation and gene expression in HCC also using the TCGA database [30]. As their analysis did not adopt inverse correlation between expression and DNA methylation, they assigned different genes from those we identified in the present study. FANCB, one of the Fanconi anemia proteins, is involved in the repair of DNA lesions and its upregulation is suggested to be required for the survival of colon cancer [31]. FANCB is also reported to be associated with other types of cancers [32, 33]. KIF family proteins, including KIF4A and KIF15 encode kinesin-related proteins which are molecular motor proteins that travel along microtubule tracks, play multiple roles in intracellular transport and cell division [34]. Kinesins are reported to have oncogenic functions such as progression and development of cancers [35]. Knockdown using siRNA and overexpression of *KIF4A* resulted in attenuation and promotion of proliferation of HCC cell lines, respectively [36]. Knockdown of *KIF15* by shRNA suppressed proliferation of HCC cell lines in vitro and in mice [37]. Recent studies reported that an increased expression of *KIF4A* and *KIF15* are potential prognostic factors in prostate cancer [38] and lung adenocarcinoma [39], respectively. *ERCC6L* encodes a newly discovered DNA helicase that is highly expressed in almost all cancers [40]. *ERCC6L* is known to be an oncogenic protein of solid tumors, since the high expression leads to cancer cell proliferation and tumor growth [40]. *ERCC6L* knockdown was demonstrated to result in downregulation of PLK1 which serves an important role in the control of the proliferation and cell cycle in cancer cells [41]. *UBE2C* encodes a member of the E2 ubiquitin-conjugating enzyme family and is required for the destruction of mitotic cyclin and for cell cycle progression. *UBE2C* expression is upregulated in various cancers including the liver [42] and abnormal expression of *UBE2C* promotes cell cycle progression [43]. The deletion of UBE2C notably reduced the level of phosphorylated aurora kinase A via Wnt/β–catenin and PI3K/Akt and results in inhibition of the cancer growth and metastasis [44]. In addition, this gene is the target of miRNAs leading to the inhibition of cancer cell growth and survival in vitro and in vivo [44].

The present study showed promoter hypomethylation of these genes is associated with increased expression in HCC patients. Newly developed technologies using genome editing, such as CRISPR/Cas9, have enabled not only site-specific DNA demethylation but also methylation in vitro and even in vivo [17–19]. Thus, manipulations of the altered methylation sites of these genes might be promising targets of HCC therapy. As described above, the five genes (*FANCB*, *KIF15*, *KIF4A*, *ERCC6L*, and *UBE2C*) are all involved in fundamental cellular functions and found in many types of cancers. Thus, manipulations of the altered methylation sites of these genes might be promising targets of therapy of HCC and possibly of other types of cancers. The relations between methylation changes of specific gene promoters and cancer etiology are yet to be investigated.

Silencing of the tumor suppressor gene *P16* by DNA methylation is known to lead to development of cancer cells [45]. The analysis of the TCGA database in the present study, however, indicated upregulation of *P16* in many types of cancers (Fig. 6f). *P16* takes a part as an early gatekeeper against cancer and the silencing begins at preinvasive stages of a variety of cancers [10, 46]. On the other hand, recent studies reported that cellular senescence, an irreversible cell cycle arrest, becomes rather a promoting factor of cancer exacerbation through acquisition of the senescence-associated secretory phenotype (SASP) and P*16* upregulation associates with cellular senescence [47–49]. Upregulation of *P16* in many types of cancers (Fig. 6f) may reflect the stage when *P16* downregulation is no longer a factor of cancer progression but rather *P16* associates with cellular senescence.

## Conclusion

In the present study, we clarified that expression of *FANCB*, *KIF15*, *KIF4A*, *ERCC6L*, and *UBE2C* are regulated by DNA methylation changes of their promoter CpGis and closely associated with tumor prognosis in HCC using the TCGA database. The manipulation of these methylations is a promising novel approach for treatment of HCC and possibly of other cancers with higher site specificity and less side effects. Furthermore, the protocol that we presented in the present study is applicable to other cancers and has great potential to drive new strategies of epigenetic cancer treatment forward.

## Supplementary information

**Additional file 1: Table S1.** Sample ID of HCC and normal liver tissues in TCGA database

**Additional file 2: Table S2.** List of up/down regulated genes in HCC tissues compared with normal liver tissues

## Data Availability

The datasets analyzed in the current study are available from the Cancer Genome Atlas (TCGA) database.

## Abbreviations

HCC: Hepatocellular carcinoma
ncRNA: non-coding RNA
CpGi: CpG island
TSS: Transcription start site
5-aza-dC: 5-aza-2′-deoxycytidine
TCGA: The Cancer Genome Atlas
GO: Gene Ontology
